# Negative and Positive Affect Regulation in a Transdiagnostic Internet-Based Protocol for Emotional Disorders: Randomized Controlled Trial

**DOI:** 10.2196/21335

**Published:** 2021-02-01

**Authors:** Amanda Díaz-García, Alberto González-Robles, Azucena García-Palacios, Javier Fernández-Álvarez, Diana Castilla, Juana María Bretón, Rosa María Baños, Soledad Quero, Cristina Botella

**Affiliations:** 1 Department of Psychology and Sociology Universidad de Zaragoza Teruel Spain; 2 Universitat Jaume I Castellón de la Plana Spain; 3 CIBER Fisiopatología Obesidad y Nutrición (CIBERObn) Instituto Carlos III Madrid Spain; 4 Department of Psychology Università Cattolica del Sacro Cuore Milan Italy; 5 Department of Personality, Evaluation and Psychological Treatments Universidad de Valencia Valencia Spain

**Keywords:** transdiagnostic, positive affectivity, negative affectivity, emotion regulation, emotional disorders, internet

## Abstract

**Background:**

Emotional disorders (EDs) are among the most prevalent mental disorders. Existing evidence-based psychological treatments are not sufficient to reduce the disease burden of mental disorders. It is therefore essential to implement innovative solutions to achieve a successful dissemination of psychological treatment protocols, and in this regard, the use of information and communication technologies such as the internet can be very useful. Furthermore, the literature suggests that not everyone with an ED receives the appropriate treatment. This situation has led to the development of new intervention proposals based on the transdiagnostic perspective, which attempts to address the underlying processes common to EDs. Most of these transdiagnostic interventions focus primarily on downregulating negative affectivity (NA), and less attention has been paid to strengths and the upregulation of positive affectivity, despite its importance for well-being and mental health.

**Objective:**

This study aims to evaluate the efficacy of a transdiagnostic internet-based treatment for EDs in a community sample.

**Methods:**

A 3-armed randomized controlled trial was conducted. A total of 216 participants were randomly assigned to a transdiagnostic internet-based protocol (TIBP), a TIBP+ positive affect (PA) component, or a waiting list (WL) control group. The treatment protocol contained core components mainly addressed to downregulate NA (ie, present-focused emotional awareness and acceptance, cognitive flexibility, behavioral and emotional avoidance patterns, and interoceptive and situational exposure) as well as a PA regulation component to promote psychological strengths and enhance well-being. Data on depression, anxiety, quality of life, neuroticism and extraversion, and PA/NA before and after treatment were analyzed. Expectations and opinions of treatment were also analyzed.

**Results:**

Within-group comparisons indicated significant pre-post reductions in the two experimental conditions. In the TIBP+PA condition, the effect sizes were large for all primary outcomes (*d*=1.42, Beck Depression Inventory [BDI-II]; *d*=0.91, Beck Anxiety Inventory [BAI]; *d*=1.27, Positive and Negative Affect Schedule-Positive [PANAS-P]; *d*=1.26, Positive and Negative Affect Schedule-Negative [PANAS-N]), whereas the TIBP condition yielded large effect sizes for BDI-II (*d*=1.19) and PANAS-N (*d*=1.28) and medium effect sizes for BAI (*d*=0.63) and PANAS-P (*d*=0.69). Between-group comparisons revealed that participants who received one of the two active treatments scored better at posttreatment than WL participants. Although there were no statistically significant differences between the two intervention groups on the PA measure, effect sizes were consistently larger in the TIBP+PA condition than in the standard transdiagnostic protocol.

**Conclusions:**

Overall, the findings indicate that EDs can be effectively treated with a transdiagnostic intervention via the internet, as significant improvements in depression, anxiety, and quality of life measures were observed. Regarding PA measures, promising effects were found, but more research is needed to study the role of PA as a therapeutic component.

**Trial Registration:**

ClinicalTrials.gov NCT02578758; https://clinicaltrials.gov/ct2/show/NCT02578758

**International Registered Report Identifier (IRRID):**

RR2-10.1186/s12888-017-1297-z

## Introduction

### Transdiagnostic Treatments for the Common Psychopathological Processes Underlying Emotional Disorders

Emotional disorders (EDs) are defined as anxiety and unipolar mood disorders. These disorders have been grouped based on their common biological and psychological vulnerabilities [1]. The estimated lifetime prevalence rates for EDs are high (28.8% for anxiety disorders and 20.8% for mood disorders). In addition, the co-occurrence of multiple EDs has also been found to be elevated, with studies showing that more than 40% of people with one diagnosis also met the diagnostic criteria for a second disorder over a 12-month period [2].

In recent years, research has demonstrated that evidence-based psychological treatments (EBTs) are effective in the treatment of EDs [3]. However, there has been little success in decreasing the prevalence and incidence of mental illness, and only a small proportion of people in need actually receive adequate psychological treatment [4]. In addition, disseminating EBTs has become a real challenge because of their cost, the duration of the treatments, and the lack of well-qualified professionals [5], which can explain why EBTs are underutilized in clinical practice settings [6].

Recently, transdiagnostic approaches have emerged that address the common characteristics found in cognitive, behavioral, emotional, and other dysregulation areas underlying different EDs, that is, the biological and psychological vulnerabilities shared by different mental disorders [7,8]. With regard to transdiagnostic processes, maladaptive emotion regulation strategies have been suggested as potential explanatory factors underlying the comorbidity across EDs [9].

In response to transdiagnostic approaches, several transdiagnostic treatments have been developed to provide patients with a set of skills geared specifically toward common vulnerabilities [10]. One example of these treatments is the Unified Protocol (UP) [7], which was designed to be applicable across different EDs and represented a significant shift toward transdiagnostic psychological treatments for EDs [11,12]. The UP has been tested and results indicate that it is effective in reducing negative affect (NA) [13], with improvements maintained at the 18-month follow-up [14]. Furthermore, the effect of the UP has been shown on the two temperament dimensions of neuroticism (N)/behavioral inhibition (BI) and extraversion (E)/behavioral activation (BA) [15].

Mounting evidence demonstrates the efficacy of transdiagnostic treatments in patients with EDs compared with control groups [16-19], showing that transdiagnostic treatments are just as effective as disorder-specific cognitive behavioral therapy (CBT) [12,20]. The data suggest that a transdiagnostic treatment for EDs might be more widely effective across a diverse range of mental disorders, addressing different disorders with a single protocol [21]. More specifically, a recent meta-analysis showed that the UP is more effective compared with different control groups, such as treatment as usual, waitlist, and medication control groups, in treating anxiety and depressive symptoms [22].

### The Role of Positive Affect in EDs

Regarding the temperamental vulnerabilities, some authors have identified two essential dimensions of temperament in the etiology and course of EDs: N/NA and E/positive affect (PA) [8]. Hence, neuroticism has been identified as a core factor involved in the development of EDs [23]. In addition, N/NA and E/PA have been closely related to Gray’s (1987) constructs of BI and BA, respectively [24-26], and these terms are often used interchangeably as the most stable measures of temperament [8,15,26,27]. Thus, people with EDs have higher levels of N/NA/BI [8], and they experience negative emotions more intensely and frequently [28] than people who do not have any ED. In contrast, the dimension of positive emotionality, E/PA/BA, has also been observed in many disorders, suggesting that people with an ED show low levels of E/BA [29], which can predict the onset of depression [30] and increase the severity of the problem [31]. Despite the importance of PA in health and well-being, there is limited research on its promotion; therefore, more research is needed in this area. Furthermore, notwithstanding the recent upsurge in transdiagnostic treatments for EDs, most of these protocols have focused on reducing NA. They have addressed core psychopathological deficits in the way patients experience and respond to negative emotions [32]. However, less attention has been paid to positive emotions or promoting PA [33]. In addition to being involved in the symptomatology of EDs, positive emotionality is considered a core element of mental health, showing beneficial, generalized effects on health and functioning [34-37]. Thus, the relationship between emotion regulation (eg, cognitive reappraisal) and well-being has also been demonstrated [38]. On the basis of the literature that highlights the potential importance of positive emotionality as a treatment component [39-43], it is necessary to develop and test treatment components focused on upregulating PA.

### Internet-Based Treatments

There are many models for delivering interventions in novel ways that can be scaled up to reach large numbers of people in need [4]. In this regard, information and communication technologies (ICTs) play an important role and can facilitate the availability of EBTs [44]. Specifically, the internet is used for the assessment and treatment of clinical conditions, and it has been established as a useful and effective tool for delivering psychological treatments to treat several psychological disorders [45], particularly depression and anxiety disorders [46]. Moreover, some meta-analyses have revealed that these interventions are as efficacious as face-to-face traditional treatments [47,48].

### This Study

The purpose of this study is to test the efficacy of a web-based psychological treatment protocol for individuals from a community sample with one or more diagnoses of EDs: major depressive disorder (MDD), dysthymic disorder (DD), obsessive-compulsive disorder, and four anxiety disorders: panic disorder (PD), agoraphobia (AG), generalized anxiety disorder (GAD), social anxiety disorder (SAD), anxiety disorder not otherwise specified, and (unipolar) mood disorder not otherwise specified [49]. Rather than focusing solely on NA, the treatment protocol includes 2 types of components: one based on classical perspectives for downregulating NA and the other aimed at upregulating PA. The protocol can be applied either in its traditional format (transdiagnostic internet-based protocol, TIBP) or by including both of these components (TIBP+PA).

Some studies have tested the efficacy of transdiagnostic interventions in improving PA measures. However, these studies do not include a specific component to address PA regulation [13], or they are uncontrolled trials [50-52]. Only one study that evaluated the efficacy of a new transdiagnostic treatment focuses on PA, but it is a pilot study rather than a randomized controlled trial (RCT) [53]. To the best of our knowledge, no published RCT has tested the efficacy of a transdiagnostic internet-based treatment for EDs with a specific component to address PA regulation. Therefore, the aim of this study is to investigate the effectiveness of this transdiagnostic protocol for EDs, with and without the specific component to upregulate PA, versus a wait-list control group. A secondary aim is to test the differential effect of the specific treatment component designed to upregulate PA. Finally, we study patients’ acceptance of the program developed to apply the treatment protocol over the internet with minimal support by the clinician. We hypothesized that (1) both self-applied protocol modalities (TIBP and TIBP+PA) would be more effective than the wait-list control condition in the treatment of EDs; (2) both interventions would result in significant improvements in depressive and anxious symptomatology at posttreatment; (3) the TIBP+PA would significantly outperform the TIBP group on PA measures; and (4) both protocols are well accepted, with no statistical differences between conditions.

## Methods

### Study Design

This study was a three-armed superiority RCT in which participants were randomly allocated to 1 of 3 conditions: (1) TIBP, (2) TIBP+PA, and (3) waiting list (WL) control condition. For ethical reasons, participants in the control condition were offered the possibility of receiving the treatment protocol after spending time on the WL (16 weeks), thus leaving no control group for the follow-up measurements. Block randomization was performed to ensure that all primary diagnoses were equally represented across conditions. The trial was registered at ClinicalTrial.gov as NCT02578758 on October 16, 2015. The study was approved by the Ethics Committee of Universitat Jaume I (Castellón, Spain; May 5, 2016) and was conducted in compliance with the study protocol, following the Consolidated Standards of Reporting Trials (CONSORT) statement [54], the CONSORT-eHealth guidelines [55], and the Standard Protocol Items: Recommendations for Interventional Trials guidelines [56,57]. Details of the study protocol have been reported elsewhere [58]. Different effect sizes found in the literature based on the transdiagnostic perspective of EDs were considered to estimate the study power in this study. These calculations were performed with the software program G*Power 3.1 [59] and published in the study protocol [58]. This study reports on pre- to posttreatment data.

### Study Population, Recruitment, and Eligibility Criteria

The clinical trial was conducted in a community sample of individuals diagnosed with one or more of the aforementioned disorders. Participants were recruited from adult volunteers interested in participating in the study between June 2015 and July 2018. Potential participants were attended by phone by the clinical team members (who had at least a university master’s degree in general health psychology) to explain the study and clarify any doubts. People interested in participating signed the web-based informed consent form and were assessed taking into account all the inclusion criteria. The inclusion criteria were as follows: (1) being at least 18 years old; (2) meeting the Diagnostic and Statistical Manual of Mental Disorders, Fourth Edition (DSM-IV) diagnostic criteria for EDs; (3) having the ability to understand and read Spanish; (4) having access to the internet and an email address; and (5) providing web-based informed consent. The exclusion criteria were as follows: (1) having schizophrenia, bipolar disorder, or alcohol and/or substance dependence disorder; (2) presence of a high risk of suicide (defined by the Mini-International Neuropsychiatry Interview [60] as greater than or equal to 10 points); (3) presence of medical disease/condition that prevents the participant from carrying out the psychological treatment; and (4) receiving another psychological treatment during the study. Receiving pharmacological treatment was not an exclusion criterion, but any increase and/or change in the medication (in the case of receiving) during the study period implied the participant’s exclusion from subsequent analyses. Participants who fulfilled all the study criteria were randomized to one of the three experimental conditions by an independent researcher. This researcher was unaware of the characteristics of the study and had no clinical involvement in the trial or access to the study data. Participants agreed to participate before determining which treatment they were allocated. All participants were free to withdraw from the treatment at any time. Access and participation in the study did not involve payment in any case.

### The Transdiagnostic Interventions

The treatment protocol is based on the transdiagnostic perspective derived from the UP [5,7] and some strategies from Marsha Linehan’s protocol [61]. Initially, a manualized protocol was developed and structured in a patient and therapist handbook. Later, the protocol was adapted to a multimedia web platform (videos, vignettes, audios, images, etc) to be completely self-applied via the internet [62] through a PC or a tablet. The ease of use of the program has been strengthened because it presents a linear navigation to optimize the treatment structure and make the treatment easier and more attractive to the participants.

The program consists of an assessment protocol and a treatment protocol that includes core components, mainly designed to downregulate NA (present-focused emotional awareness and acceptance, cognitive flexibility, behavioral and emotional avoidance patterns, and interoceptive and situational exposure) and upregulate PA to promote psychological strengths and enhance well-being [63]. The protocol content is adapted from the UP [7] and some of the strategies for emotion regulation from dialectical behavior therapy [61]. The PA regulation component is based mainly on BA strategies [64], strategies to promote pleasant and significant activities linked to values and life goals, and strategies to enhance personal strengths, positive feelings, positive cognitions, and positive behavior [63,65]. Furthermore, well-being therapy strategies [66,67] and some concepts from Fredickson’s Broaden-and-Build Theory [68] are also included in the program. The PA regulation component takes place after the NA regulation component. The protocol also includes traditional therapeutic components of evidence-based treatment for ED (psychoeducation, motivation for change, and relapse prevention). All the treatment components were developed through two self-applied protocol modalities (TIBP and TIBP+PA) with 12 and 16 modules, respectively, with the only difference being the inclusion or absence of the modules that contain the PA-regulation component. A detailed description of modules that contain the PA regulation component is presented in Multimedia Appendix 1 [64,66,68-77]. The modules in each intervention protocol are described briefly elsewhere [58].

The duration of the program could vary among users, and participants in both treatment conditions had equal access to the protocol for a maximum period of 18 weeks. The program sent weekly messages to the patient to remind him/her to continue to work to benefit from the program. A professional platform was used to send these messages [78]. The program also sent automatic emails with reminders to access the modules when participants had not entered the past 15 days. In addition to this ICT support, human support was also provided through weekly phone calls (maximum of 5 min) during the treatment period to resolve any difficulties or doubts, or to remind them of the importance of reviewing the treatment contents.

### Outcome Measures

The assessment protocol was included at the beginning and end of the web-based program. A detailed description of the measures and their aims has been published elsewhere [58]. The measures included in this study are described in Table 1.

**Table 1:** Study measures.

Measure				Aim		Cronbach α		ω^a^		Time of assessment
**Diagnostic interview**										
	MINI^b^		Psychiatric diagnosis		N/A^c^		N/A		BL^d^	
**Primary outcomes**										
	BDI-II^e^		Severity of depression		.91		0.91		BL, Post-T^f^	
	BAI^g^		Severity of anxiety		.92		0.92		BL, Post-T	
	PANAS^h^		Positive and negative affect		PA^i^=.91; NA^j^=.89		PA=0.91; NA=0.89		BL, Post-T	
**Secondary outcomes**										
	**Personality measures**									
		NEO FFI^k^	Neuroticism and extraversion		N^l^=.81; E^m^=.84		N=0.82; E=0.84		BL, Post-T	
	**Quality of life**									
		EQ-5D^n^	Health-related quality of life		.67		0.70		BL, Post-T	
	**Expectation and opinion**									
		Expectation of treatment scale	Expectation of treatment		N/A		N/A		BL	
		Opinion of treatment scale	Opinion of treatment		N/A		N/A		Post-T	

^a^ω: coefficient omega in this study.

^b^MINI: Mini-International Neuropsychiatric Interview, Version 5.0.0.

^c^N/A: not applicable.

^d^BL: baseline.

^e^BDI-II: Beck Depression Inventory-II.

^f^Post-T: posttreatment.

^g^BAI: Beck Anxiety Inventory.

^h^PANAS: Positive and Negative Affect Schedule.

^i^PA: positive affect.

^j^NA: negative affect.

^k^NEO FFI: NEO Five Factor Inventory.

^l^N: neuroticism.

^m^E: extraversion.

^n^EQ-5D: EuroQoL-5D Questionnaire.

### Statistical Analysis

Group differences in participants’ sociodemographic and clinical data at baseline were examined to confirm that they were comparable after randomization. One-way analysis of variance for continuous variables and Fisher exact tests of independence for categorical variables were used. Intention-to-treat (ITT) using mixed models, with full information maximum likelihood estimation and without any ad hoc imputations were conducted to handle missing data due to participant dropout [79]. This approach uses all available data, does not substitute missing values with assumed or estimated values, and does not assume that the last measurement is stable (the last observation carried forward assumption) [80]. Mixed model analyses are appropriate for RCTs with multiple time points and pre-to postonly designs with substantial dropout rates [81]. The pattern of missingness was investigated to determine its likelihood of being random rather than systematic (missing not at random, MNAR). Subsequently, associations between sample characteristics missingness in the outcome variables were examined (*t* tests for continuous variables and Fisher exact tests for categorical variables). A linear mixed model for each outcome measure was implemented using the linear mixed-effects models (MIXED) procedure with one random intercept per subject. An identity covariance structure was specified to model the covariance structure of the random intercept. Significant effects were followed up with pairwise comparisons using the Bonferroni correction. Effect sizes were calculated for within- and between-group comparisons using the standardized observed mean difference proposed by Cohen [82]. To determine the existence of a reliable change in a patient, the reliable change index (RCI; Jacobson and Truax’s method) [83] was used. The RCI values for the primary outcomes (Beck Depression Inventory, BDI-II; Beck Anxiety Inventory, BAI; Positive and Negative Affect Schedule-Positive [PANAS-P]; and Positive and Negative Affect Schedule-Negative [PANAS-N]) were calculated for the completer sample (participants who provided data at posttreatment). Fisher exact tests were performed to evaluate group differences in RCI rates for completers. All statistical analyses were conducted using IBM SPSS Statistics for Windows, version 22, and SAS software, version 9.4, of the SAS System for Windows.

## Results

### Participant Flow and Attrition

**Figure 1:**
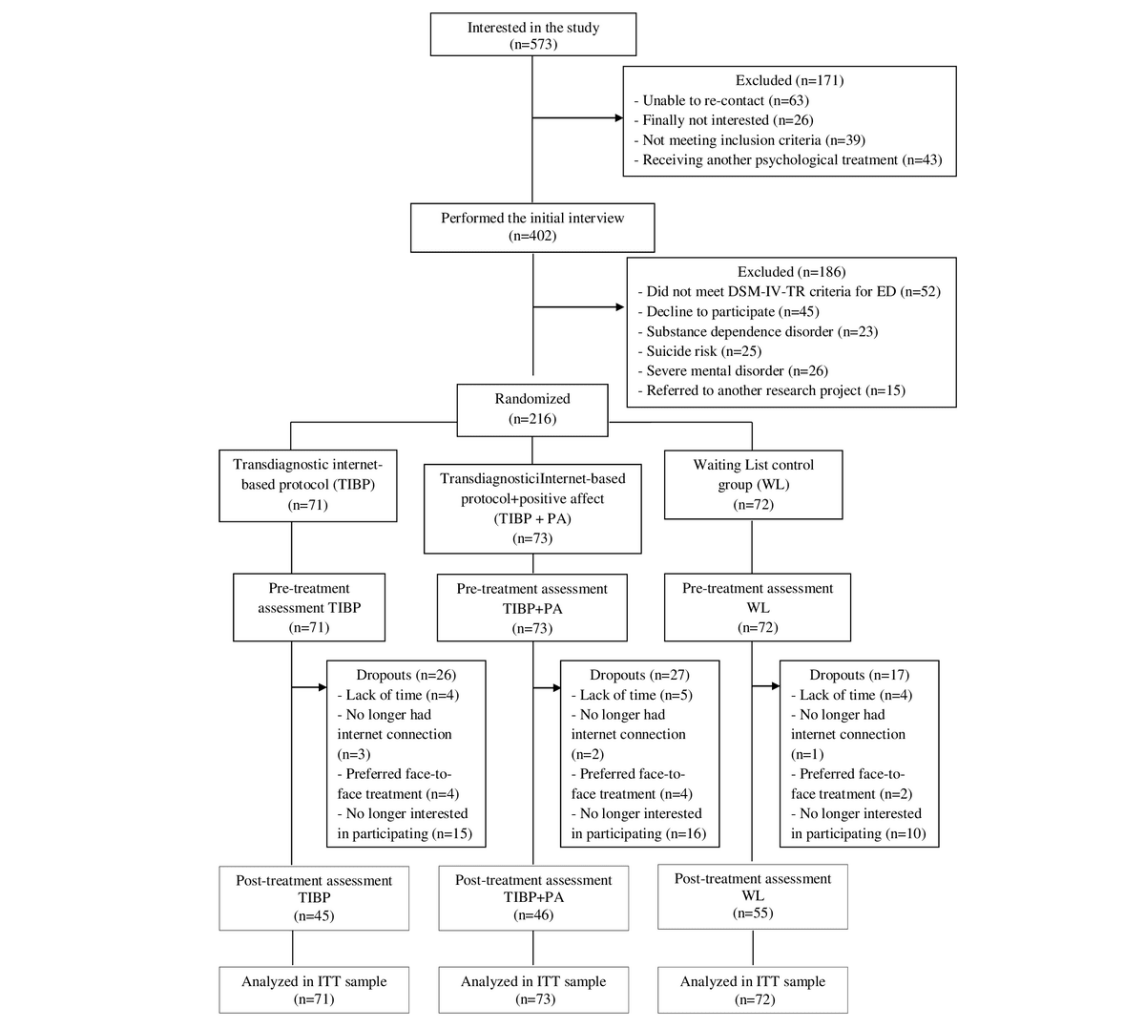
Flowchart of participants. DSM-IV-TR: Diagnostic and Statistical Manual of Mental Disorders, Fourth Edition, Text Revision; ED: emotional disorder; TIBP: Transdiagnostic internet-Based Protocol; PA: positive affect; WL: Waiting List; ITT: intention-to-treat.

Out of the 573 people who expressed initial interest in the study, as the flow diagram shows (Figure 1), only 402 performed the initial interview. At this stage, 186 participants failed to meet the inclusion criteria. Finally, 216 patients were included in the study, and they were randomly allocated to each experimental condition: TIBP, n=71; TIBP+PA, n=73; WL, n=72. Regarding pretreatment assessments, 71 participants performed it in the TIBP, 73 in the TIBP+PA, and 72 in the WL. A similar number of participants performed the posttreatment assessment from both intervention conditions (TIBP, n=45; TIBP+PA, n=46). No significant differences between the three conditions were found in dropout rates (X^2^_2_=3.8, *P*=.14). In the TIBP condition, of those who started the program (n=71), 26 participants (26/71, 37%) withdrew from the treatment. In the TIBP+PA condition, a similar pattern was found; of those who started the program (n=73), 27 participants (27/73, 37%) withdrew from the treatment. Finally, in the WL control group, data from 55 participants were obtained after they had spent 16 weeks on the WL (55/72, 76% retention; 17/72, 24% dropout). Overall, of the 216 participants who started the study, 86 participants withdrew from the program. As a result, two patterns of missingness emerged. One of them represented 32% of the sample (70/216, 32.4%) and the other represented a very low percentage (16/216, 7.4%). Missingness was not related to the characteristics listed in Table 2 in any of the three arms of the RCT (all *P*>.05 in both patterns). Therefore, patterns of missingness were not found to be MNAR and the decision to continue the analysis with the available data was made [84,85].

**Table 2:** Demographic characteristics of participants at pre-assessment (N=216).

Variable			TIBP^a^ (n=71)		TIBP+PA^b^ (n=73)		WL^c^ (n=72)		Total (N=216)		Statistic^d^		*P* value	
Age (years), mean (SD); range			35.82 (13.04); 18-72		33.11 (9.74); 19-52		31.82 (10.50); 19-58		33.57 (11.24); 18-72		*F*_2213_=2.382		.09	
**Sex, n (%)**												*X*_2_^2^=0.2		.88
	Female		51 (72)		51 (70)		53 (74)		155 (71.8)					
	Male		20 (28)		22 (30)		19 (26)		61 (28.2)					
**Marital status, n (%)**												N/A^e^		.97
	Single		44 (62)		41 (56)		42 (58)		127 (58.8)					
	Married or partnered		23 (32)		27 (37)		25 (35)		75 (34.7)					
	Divorced or widowed		4 (6)		5 (7)		5 (7)		14 (6.5)					
**Education level, n (%)**												N/A		.46
	Basic studies		1 (1)		5 (7)		3 (4)		9 (4.2)					
	Medium studies		15 (21)		12 (16)		17 (24)		44 (20.4)					
	Higher studies		55 (78)		56 (77)		52 (72)		163 (75.5)					
**Principal diagnosis, n (%)**												N/A		.57
	MDD^f^		11 (15)		9 (12)		16 (22)		36 (16.7)					
	DD^g^		1 (1)		0 (0)		2 (3)		3 (1.4)					
	GAD^h^		26 (37)		21 (29)		24 (33)		71 (32.9)					
	PD^i^/AG^j^		4 (6)		6 (8)		6 (8)		16 (7.4)					
	PD		2 (3)		4 (6)		3 (4)		9 (4.2)					
	AG		6 (9)		6 (8)		1 (1)		13 (6.0)					
	SAD^k^		15 (21)		23 (32)		16 (22)		54 (25.0)					
	OCD^l^		3 (4)		1 (1)		2 (3)		6 (2.8)					
	Anxiety NOS^m^		3 (4)		3 (4)		1 (1)		7 (3.2)					
	Depression NOS		0 (0)		0 (0)		1 (1)		1 (0.5)					
**Number of comorbid disorders, n (%)**												N/A		.17
	0		32 (45)		19 (26)		34 (47)		81 (37.5)					
	**1**		28 (39)		36 (49)		26 (36)		92 (42.6)					
		MDD		18 (64)		22 (61)		15 (57)		57 (62)				
		DD		1 (4)		2 (6)		3 (12)		6 (7)				
		GAD		2 (7)		7 (19)		2 (8)		11 (12)				
		PD/AG		0 (0)		0 (0)		0 (0)		0 (0.0)				
		PD		0 (0)		0 (0)		1 (4)		1 (1)				
		AG		3 (11)		2 (6)		2 (8)		7 (8)				
		SAD		4 (14)		3 (8)		3 (11)		10 (10)				
		OCD		0 (0)		0 (0)		0 (0)		0 (0)				
		Anxiety NOS		0 (0)		0 (0)		0 (0)		0 (0)				
		Depression NOS		0 (0)		0 (0)		0 (0)		0 (0)				
	**2**		7 (10)		12 (16)		7 (10)		27 (12.5)					
		MDD and GAD		2 (29)		3 (25)		2 (29)		7 (26)				
		MDD and PD/AG		1 (14)		1 (8)		1 (14)		3 (11)				
		MDD and PD		1 (14)		1 (8)		2 (29)		4 (15)				
		MDD and AG		0 (0)		2 (17)		0 (0)		3 (11)				
		MDD and OCD		1 (14)		0 (0)		0 (0)		1 (4)				
		DD and SAD		0 (0)		1 (8)		0 (0)		1 (4)				
		GAD and SAD		2 (29)		1 (8)		1 (14)		4 (15)				
		GAD and AG		0 (0)		2 (17)		0 (0)		2 (7)				
		PD/AG and SAD		0 (0)		0 (0)		1 (14)		1 (4)				
		SAD and OCD		0 (0)		1 (8)		0 (0)		1 (4)				
	**3**		4 (6)		6 (8)		5 (7)		16 (7.4)					
		MDD, GAD, and AG		1 (25)		1 (17)		2 (40)		4 (25)				
		MDD, GAD, and SAD		0 (0)		2 (33)		2 (40)		4 (25)				
		MDD, GAD, and OCD		0 (0)		1 (17)		0 (0)		2 (13)				
		MDD, PD, and SAD		0 (0)		2 (33)		0 (0)		2 (13)				
		MDD, PD, and OCD		0 (0)		0 (0)		1 (20)		1 (6)				
		MDD, AG, and SAD		1 (25)		0 (0)		0 (0)		1 (6)				
		MDD, SAD, and OCD		1 (25)		0 (0)		0 (0)		1 (6)				
		GAD, PD/AG, and SAD		1 (25)		0 (0)		0 (0)		1 (6)				

^a^TIBP: transdiagnostic internet-based protocol.

^b^TIBP+PA: transdiagnostic internet-based protocol+positive affect component.

^c^WL: waiting list.

^d^Statistic: Pearson chi-square or Fisher exact test.

^e^N/A: not applicable.

^f^MDD: major depressive disorder.

^g^DD: dysthymic disorder.

^h^GAD: generalized anxiety disorder.

^i^PD: panic disorder.

^j^AG: agoraphobia.

^k^SAD: social anxiety disorder.

^l^OCD: obsessive-compulsive disorder.

^m^NOS: not otherwise specified.

### Baseline Data and Participant Characteristics

Details about participants’ sociodemographic characteristics for each group at pretreatment are presented in Table 2. The results indicated that there were no significant differences between the experimental groups before treatment for any of these variables, indicating that the randomization was successful. Overall, participants’ mean age was 33.57 years (SD 11.24, range 18-72), the majority were females (155/216, 71.8%), and most of them were single (127/216, 58.8%) and had completed or were pursuing higher studies (163/216, 75.5%; eg, undergraduate degree studies, graduate studies or university master’s degrees, or postgraduate studies or doctoral degrees). Principal and comorbid diagnoses are presented in Table 2. Most of the participants had GAD (71/216, 32.9%), followed by SAD (54/216, 25.0%) and MDD (36/216, 16.7%). Regarding the patterns of comorbidity in the sample, 41.7% (90/216) of the participants had at least one comorbid diagnosis, with MDD being the most common comorbid disorder (n=57), followed by GAD (n=11), SAD (n=10), AG (n=7), DD (n=6), and PD (n=1).

Regarding the clinical characteristics of the participants in each experimental condition at pretreatment (Table 3), no statistically significant differences were found between the groups on any of the primary and secondary outcomes.

**Table 3:** Clinical characteristics of participants at pre-assessment.

Measure			TIBP^a^ (n=71), mean (SD)	TIBP+PA^b^ (n=73), mean (SD)	WL^c^ (n=72), mean (SD)	Total (N=216), mean (SD)	Statistic, *F* (*df*)	*P* value
**Primary outcomes**								
	BDI-II^d^		25.35 (11.33)	29.07 (11.33)	26.31 (12.43)	26.93 (11.76)	1.966 (2213)	.14
	BAI^e^		21.14 (12.10)	23.58 (11.50)	21.87 (12.56)	22.23 (12.04)	0.719 (2,197)	.49
	PANAS^f^*_*Positive		20.72 (6.52)	19.32 (6.22)	19.28 (5.67)	19.84 (6.22)	1.277 (2213)	.28
	PANAS*_*Negative		30.68 (7.73)	31.96 (8.79)	28.63 (9.03)	30.43 (8.61)	2.807 (2213)	.06
**Secondary outcomes**								
	**Personality measures**							
		NEO FFI^g^_Neuroticism	31.54 (6.65)	34.11 (8.18)	31.39 (8.20)	32.36 (7.78)	2.853 (2213)	.06
		NEO FFI_Extraversion	21.49 (8.77)	19.26 (7.59)	21.79 (8.66)	20.84 (8.39)	1.991 (2213)	.14
	**Quality of life**							
		EQ-5D^h^	54.60 (17.76)	54.78 (19.75)	51.91 (17.81)	53.75 (18.44)	0.511 (2197)	.60

^a^TIBP: transdiagnostic internet-based protocol.

^b^TIBP+PA: transdiagnostic internet-based protocol+positive affect component.

^c^WL: waiting list.

^d^BDI-II: Beck Depression Inventory-II.

^e^BAI: Beck Anxiety Inventory.

^f^PANAS: Positive and Negative Affect Schedule.

^g^NEO FFI: NEO Five Factor Inventory.

^h^EQ-5D: EuroQoL-5D questionnaire.

### Effectiveness of the Intervention on Primary and Secondary Outcomes at Pre-Post

#### Primary Outcomes

Table 4 includes descriptive statistics (ie, means and SD) for TIBP+PA, TIBP, and WL at pretreatment and posttreatment; Table 5 includes within-group and between-group effect sizes and CIs for all the primary outcome measures in the three experimental groups, based on the ITT sample.

For the three primary outcomes, within-group comparisons indicated significant pre-post reductions in the two experimental conditions, with large effect sizes for the BDI-II (*d*=1.19) and PANAS-N (*d*=1.28), and moderate effect sizes for the BAI (*d*=0.63) and PANAS-P (*d*=0.69) in the TIBP condition. In the TIBP+PA condition, the effect sizes were large for all primary outcomes (*d*=1.42, BDI-II; *d*=0.91, BAI; *d*=1.27, PANAS-P; *d*=1.26, PANAS-N). Between-group comparisons revealed that participants who received the treatment scored better at posttreatment than the WL group. Greater reductions were found in the BDI-II scores in the TIBP condition than in the WL condition (mean difference −13.61; *P*<.001; *d*=1.18; 95% CI −1.61 to −0.76), as well as between the TIBP+PA condition and WL (mean difference −14.31; *P*<.001; *d*=1.05; 95% CI −1.46 to −0.63), with large effect sizes. No differences were found between the two experimental conditions (mean difference 0.70; *P*=.76; *d*=0.10; 95% CI −0.51 to −0.31).The results for BAI scores were similar to the pattern of findings for the BDI: greater reductions in the TIBP condition (mean difference −8.19; *P*=.001; *d*=0.63; 95% CI −1.07 to −0.20) and TIBP+PA condition (mean difference −9.28; *P*<.001; *d*=0.68; 95% CI −1.10 to −0.26), compared with WL, with medium effect sizes, and no differences between the two experimental conditions (mean difference 1.09; *P*=.65; *d*=0.05; 95% CI −0.39 to 0.49). Finally, patients in the TIBP condition experienced a large increase in PA (PANAS-P) compared with WL (mean difference 5.42; *P*<.001; *d*=0.74; 95% CI 0.33 to 1.15) with moderate effect sizes and greater reductions in NA (PANAS-N; mean difference −8.34; *P*<.001; *d*=0.99; 95% CI −1.41 to −0.57) compared with WL with large effect sizes. Participants in the TIBP+PA condition experienced the same pattern as the participants in the TIBP condition but achieving large effect sizes for both higher PA (mean difference 7.86; *P*<.001; *d*=0.90; 95% CI 0.49 to 1.31) and lower NA (mean difference −8.32; *P*<.001; *d*=0.91; 95% CI −1.32 to −0.50) than participants in the WL condition. No differences were found between the two experimental conditions on PA (mean difference −2.44; *P*=.08; *d*=0.25; 95% CI −0.66 to 0.17) or NA (mean difference −0.02; *P*=.99; *d*=0.01; 95% CI −0.42 to 0.40).

**Table 4:** Descriptive statistics for TIBP+PA, TIBP, and WL at pretreatment and posttreatment for primary outcomes, personality measures, and quality of life measures.

Measures			TIBP+PA^a^ (n=46), mean (SD)					TIBP^b^ (n=45), mean (SD)				WL^c^ (n=55), mean (SD)		
			Pre-T^d^		Post-T^e^		Pre-T		Post-T		Pre-T		Post-T	
**Primary outcomes**														
	BDI-II^f^		29.07 (11.33)		12.78 (10.76)		25.35 (11.33)		11.76 (9.02)		26.31 (12.42)		26.09 (13.99)	
	BAI^g^		23.58 (11.50)		12.97 (10.33)		21.14 (12.10)		13.46 (10.26)		21.87 (12.56)		21.67 (14.41)	
	Positive Affect subscale of the Positive and Negative Affect Schedule		19.32 (6.22)		27.28 (9.21)		20.72 (6.52)		25.24 (7.10)		19.28 (5.68)		19.86 (7.27)	
	Negative Affect subscale of the Positive and Negative Affect Schedule		31.96 (8.79)		20.78 (8.37)		30.68 (7.73)		20.71 (6.81)		28.63 (9.03)		28.76 (8.99)	
**Secondary outcomes**														
	**Personality measures**													
		Neuroticism subscale of the NEO FFI^h^		34.11 (8.18)		27.67 (8.37)		31.54 (6.65)		26.64 (7.83)		31.39 (8.20)		31.71 (8.56)
		Extraversion subscale of the NEO FFI		19.26 (7.59)		24.22 (7.64)		21.49 (8.77)		24.13 (8.41)		21.79 (8.66)		19.84 (10.02)
	**Quality of life**													
		EuroQoL-5D-3L questionnaire		54.78 (19.75)		68.81 (18.37)		54.60 (17.76)		64.05 (18.78)		51.91 (17.81)		52.55 (18.96)

^a^TIBP+PA: transdiagnostic internet-based protocol + positive affect component.

^b^TIBP: transdiagnostic internet-based protocol.

^c^WL: waiting list.

^d^Pre-T: Pre-treatment.

^e^Post-T: Post-treatment.

^f^BDI-II: Beck Depression Inventory-II.

^g^BAI: Beck Anxiety Inventory.

^h^NEO FFI: NEO Five Factor Inventory.

**Table 5:** Within- and between-group effect sizes and 95% CIs.

Measures				TIBP+PA^a^, *d* (95% CI)		TIBP^b^, *d* (95% CI)		WL^c^, *d* (95% CI)		TIBP+PA versus TIBP, *d* (95% CI)		TIBP+PA versus WL, *d* (95% CI)		TIBP versus WL, *d* (95% CI)
				Pre-post		Pre-post		Pre-post		Post-T^d^		Post-T		Post-T
**Primary outcomes**														
	BDI-II^e^		1.42 (1.09 to 1.76)		1.19 (0.90 to 1.48)		0.02 (−0.13 to 0.16)		−0.10 (−0.51 to 0.31)		−1.05 (−1.46 to −0.63)		−1.18 (−1.61 to −0.76)	
	BAI^f^		0.91 (0.64 to 1.18)		0.63 (0.38 to 0.87)		0.02 (−0.12 to 0.15)		0.05 (−0.39 to 0.49)		−0.68 (−1.10 to − 0.26)		−0.63 (−1.07 to −0.20)	
	Positive Affect subscale of the Positive and Negative Affect Schedule		−1.27 (−1.58 to −0.95)		−0.69 (−0.92 to −0.45)		−0.01 (−0.22 to 0.20)		−0.25 (−0.66 to 0.17)		0.90 (0.49 to 1.31)		0.74 (0.33 to 1.15)	
	Negative Affect subscale of the Positive and Negative Affect Schedule		1.26 (0.94 to 1.57)		1.28 (0.96 to 1.60)		−0.04 (−0.22 to 0.14)		−0.01 (−0.42 to 0.40)		−0.91(−1.32 to −0.50)		−0.99 (−1.41 to −0.57)	
**Secondary outcomes**														
	**Personality measures**													
		Neuroticism subscale of the NEO FFI^g^	0.78 (0.51 to 1.05)		0.73 (0.50 to 0.96)		0.04 (−0.22 to 0.20)		−0.12 (−0.54 to 0.29)		−0.47 (−0.87 to −0.08)		0.61 (−1.01 to −0.21)	
		Extraversion subscale of the NEO FFI	0.65 (−0.88 to −0.42)		0.30 (−0.45 to −0.14)		0.22 (0.07 to 0.37)		−0.01 (−0.42 to 0.40)		0.48 (0.08 to 0.88)		0.46 (0.06 to 0.86)	
	**Quality of life**													
		EuroQoL-5D-3L questionnaire	0.70 (−0.97 to −0.43)		0.53 (−0.80 to −0.25)		0.04 (−0.21 to 0.14)		−0.25 (−0.70 to 0.19)		0.86 (0.44 to 1.29)		0.60 (0.17 to 1.04)	

^a^TIBP+PA: transdiagnostic internet-based protocol+positive affect component.

^b^TIBP: transdiagnostic internet-based protocol.

^c^WL: waiting list.

^d^Post-T: posttreatment.

^e^BDI-II: Beck Depression Inventory-II.

^f^BAI: Beck Anxiety Inventory.

^g^NEO FFI: NEO Five Factor Inventory.

#### Secondary Outcomes

Table 4 includes descriptive statistics (ie, mean and SD) for TIBP+PA, TIBP, and WL at pretreatment and posttreatment, and Table 5 includes within-group and between-group effect sizes and CIs for secondary outcomes related to personality and quality of life measures in the three experimental groups based on the ITT sample.

Regarding personality measures, within-group comparisons indicated a significant pre-to-post reduction in neuroticism in the two experimental conditions, with moderate effect sizes in NEO Five Factor Inventory (NEO FFI)-Neuroticism (*d*=0.73, TIBP; *d*=0.78, TIBP+PA), and a significant pre-to-post increase in extraversion in the two experimental conditions, with a small effect size in the TIBP condition (*d*=0.30) and a moderate effect size in the TIBP+PA condition (*d*=0.65). In the WL control group, significant changes with small effect sizes were also found on NEO FFI-Extraversion (*d*=0.22). Between-group comparisons revealed that participants who received the treatment scored better at posttreatment on NEO FFI-Neuroticism in both intervention groups compared with the WL group, with moderate effect sizes (*d*=0.61, TIBP; *d*=0.47, TIBP+PA). NEO FFI-Extraversion showed better scores with small effect sizes in both intervention conditions (*d*=0.46, TIBP; *d*=0.48, TIBP+PA) than WL. No statistically significant differences were found between the two experimental conditions for the personality measures.

Finally, quality of life measures (ie, EuroQoL-5D Questionnaire; EQ-5D) showed a significant time effect (F_1,152.98_=32.98; *P*<.001). Both intervention groups experienced significant improvements in quality of life posttreatment, and this improvement was not found in the WL control group. Within-group comparisons indicated moderate effect sizes in the TIBP condition (*d*=0.53), moderate effect sizes in the TIBP+PA condition (*d*=0.70), and nonsignificant changes in the WL control group. Between-group comparisons revealed that participants who received the treatment (with or without the specific component to upregulate PA) scored higher on quality of life at posttreatment, compared with the WL, with moderate effect sizes in the TIBP condition (*d*=0.60) and large effect sizes in the TIBP+PA condition (*d*=0.86; Table 4 for details). The differences between the two treatment groups were not statistically significant.

#### Expectations and Satisfaction

Table 6 lists the results for the two interventions groups. Items for expectations and satisfaction were rated from 0 (strongly disagree) to 10 (strongly agree), covering how logical the treatment seemed, to what extent it could satisfy the patient, whether it could be used to treat other psychological problems, and its usefulness for the patient’s specific problem. Before the treatment, all the scores were high. The analysis revealed statistically significant differences between the two conditions on expectations about the treatment: before treatment, participants in the TIBP+PA condition considered the treatment more logical (F_1,89_=4.49; *P*=.03), more satisfactory (F_1,89_=6.29; *P*=.01), more recommendable to others (F_1,89_=6.15; *P*=.01), and more useful for other psychological problems (F_1,89_=7.38; *P*=.008) than the TIBP participants did. In addition, at posttreatment, participants’ satisfaction scores were also high. The analysis revealed statistically significant differences between the two conditions on satisfaction: after treatment, participants in the TIBP+PA condition considered the treatment more satisfactory (F_1,89_=4.10; *P*=.04), recommendable to others (F_1,89_=6.79; *P*=.01), more useful for other psychological problems (F_1,89_=5.13; *P*=.02), and more useful for the patient (F_1,89_=5.91; *P*=.01) than participants in the TIBP condition did.

**Table 6:** Means and SDs for expectations and satisfaction.

Statements		Expectations		Satisfaction	
		n	Mean (SD)	n	Mean (SD)
**How logical do you think this treatment is?**					
	Total sample	91	8.21 (1.55)	91	8.21 (1.67)
	TIBP^a^	45	7.87 (1.71)	45	8.07 (1.68)
	TIBP+PA^b^	46	8.54 (1.31)	46	8.35 (1.66)
**How satisfied are you with the treatment received?**					
	Total sample	91	8.21 (1.75)	91	7.63 (2.02)
	TIBP	45	7.76 (1.86)	45	7.20 (2.04)
	TIBP+PA	46	8.65 (1.54)	46	8.04 (1.93)
**To what extent do you feel confident about recommending this treatment to a friend who has the same problems?**					
	Total sample	91	8.36 (1.77)	91	8.01 (2.12)
	TIBP	45	7.91 (1.95)	45	7.44 (2.32)
	TIBP+PA	46	8.80 (1.45)	46	8.57 (1.75)
**Do you think this treatment could be useful for treating other psychological disorders?**					
	Total sample	91	8.03 (1.72)	91	7.88 (1.76)
	TIBP	45	7.56 (1.79)	45	7.47 (1.89)
	TIBP+PA	46	8.50 (1.52)	46	8.28 (1.53)
**To what extent do you think the treatment was helpful to you?**					
	Total sample	91	7.44 (1.93)	91	7.30 (2.07)
	TIBP	45	7.13 (1.87)	45	6.78 (2.00)
	TIBP+PA	46	7.74 (1.97)	46	7.80 (2.03)

^a^TIBP: transdiagnostic internet-based protocol.

^b^TIBP+PA: transdiagnostic internet-based protocol+positive affect component.

#### Reliable and Clinically Significant Change

**Figure 2:**
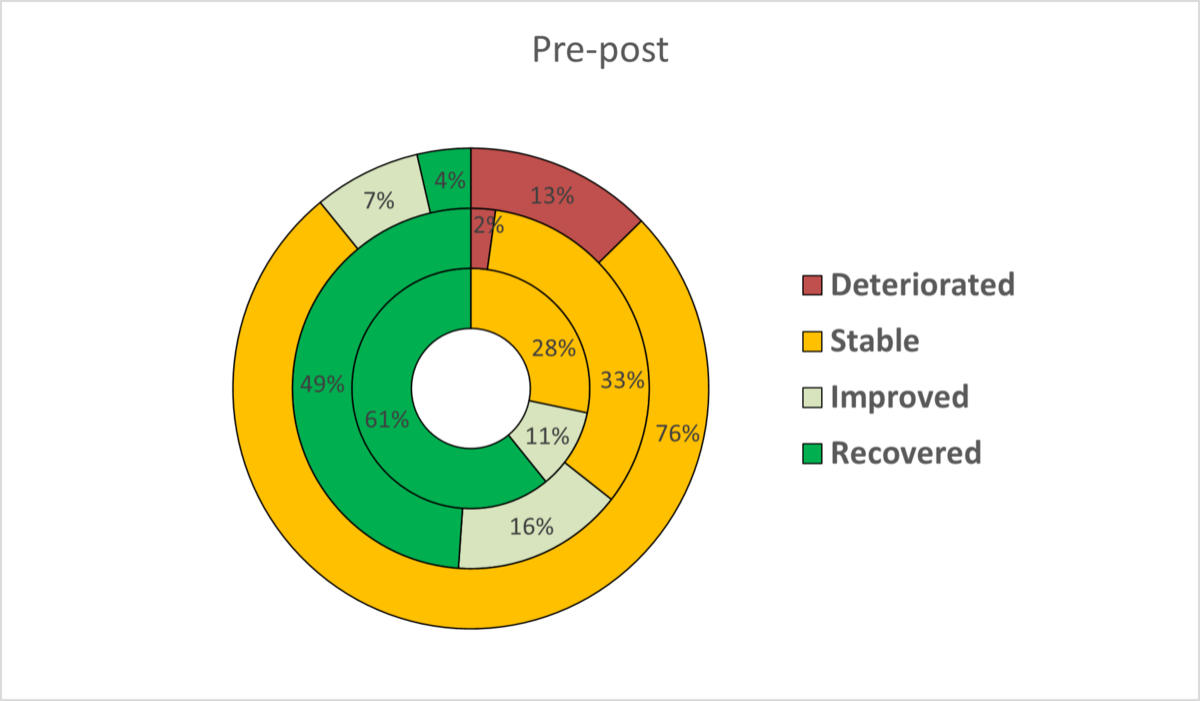
Percentages of participants recovered, improved, stable, and deteriorated on depression scores (Beck Depression Inventory-II) in transdiagnostic internet-based protocol+positive affect component (inner circle), transdiagnostic internet-based protocol (middle circle), and waiting list (outer circle).

**Figure 3:**
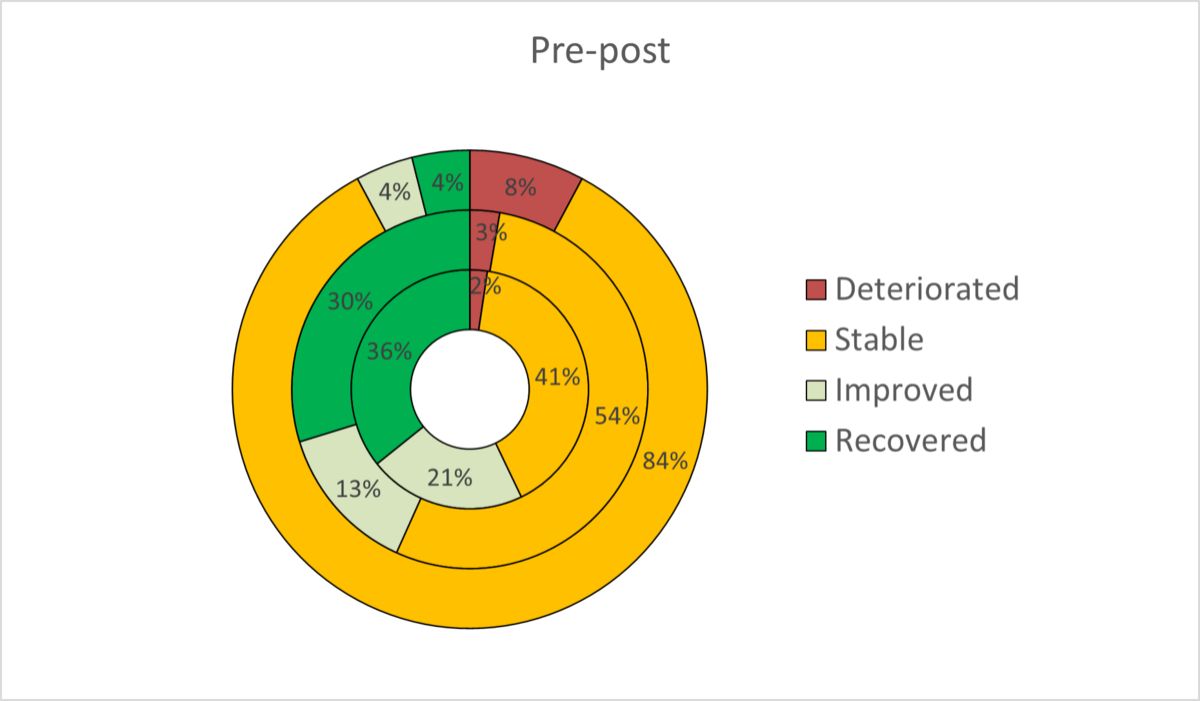
Percentages of participants recovered, improved, stable, and deteriorated on depression scores (Beck Anxiety Inventory) in transdiagnostic internet-based protocol+positive affect component (inner circle), transdiagnostic internet-based protocol (middle circle), and waiting list (outer circle).

**Figure 4:**
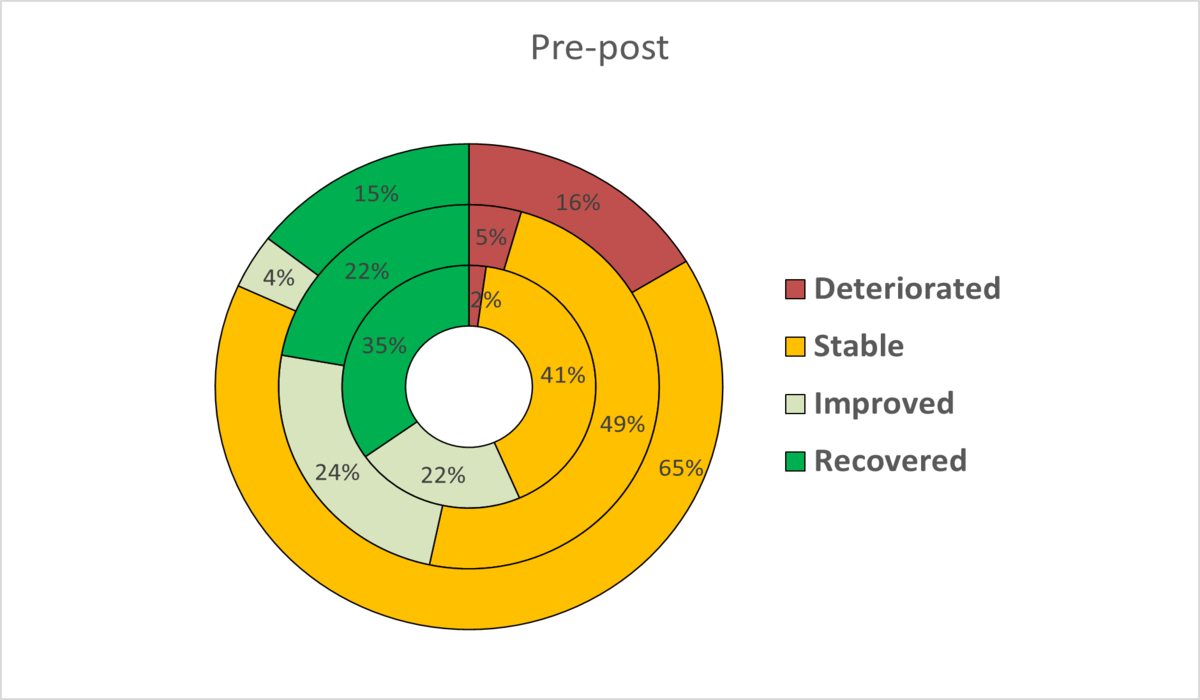
Percentages of participants recovered, improved, stable, and deteriorated on depression scores (Positive and Negative Affect Schedule_Positive subscale) in transdiagnostic internet-based protocol+positive affect component (inner circle), transdiagnostic internet-based protocol (middle circle), and waiting list (outer circle).

**Figure 5:**
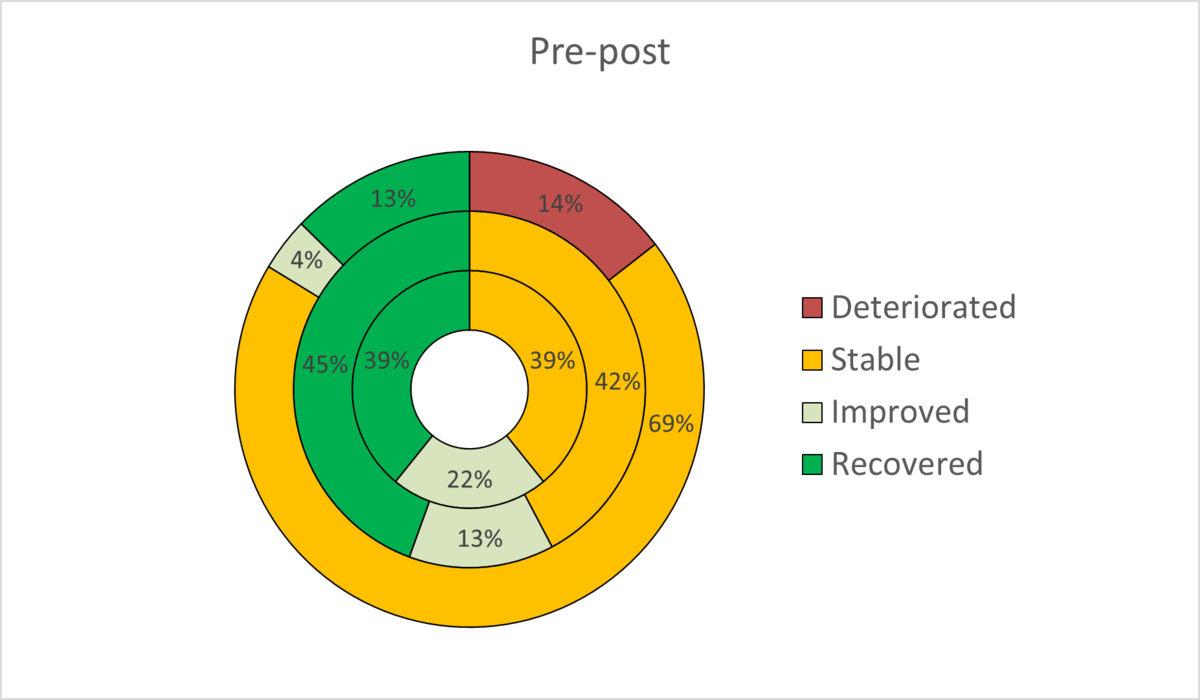
Percentages of participants recovered, improved, stable, and deteriorated on depression scores (Positive and Negative Affect Schedule_Negative subscale) in transdiagnostic internet-based protocol+positive affect component (inner circle), transdiagnostic internet-based protocol (middle circle), and waiting list (outer circle).

On the basis of the two criteria proposed by Jacobson and Truax to estimate clinically meaningful improvement, patients were classified as recovered, improved, stable, or deteriorated at posttreatment. The RCI has been expressed graphically to facilitate a more intuitive interpretation (Figures 2-5). At posttreatment, statistically significant differences were found between the three conditions in these percentages for all primary outcomes: BDI-II (*P*<.001), BAI (*P*<.001), PANAS-P (*P*=.001), and PANAS-N (*P*<.001). Overall, participants who received the transdiagnostic internet-based interventions showed a higher percentage of recovery compared with WL, which obtained high percentages of reliable deterioration on all the primary outcomes. No statistically significant differences were found between TIBP+PA and TIBP (BDI-II, *P*=.59; BAI, *P*=.67; PANAS-P, *P*=.59; and PANAS-N, *P*=.59).

## Discussion

### Principal Findings

The main objective of this study was to test the efficacy of a transdiagnostic internet-based psychological treatment protocol, with and without a specific component to upregulate PA, versus a WL control group, for individuals from a community sample with one or more ED diagnoses. The results showed that on the primary outcome measures, there was a significant time effect, with medium (BAI and PANAS-P) to large effect sizes (BDI-II and PANAS-N) in the TIBP condition and large effect sizes on all measures in the TIBP+PA condition. In the WL group, the effect size was minimal. These results are comparable with those reported in other transdiagnostic trials in which effect sizes are larger for depression than for anxiety and higher for PANAS-P than for PANAS-N [13]. A possible explanation for the larger effects for depression than for anxiety could be that the therapeutic techniques more related to the treatment of anxiety disorders (ie, consequences of emotional avoidance, emotion-driven behaviors, and interoceptive and situational exposure) are presented near the end of the treatment protocol (modules from 8 to 11). Patients are evaluated clinically shortly after these modules, and consequently, they have less time to practice them. Likewise, the effects for PA were larger in the TIBP+PA condition (*d*=1.27) than in the TIBP condition (*d*=0.69). This result is consistent with the fact that this study includes a specific component to upregulate PA, which places greater emphasis on positive experiences. One of the aims of the intervention is to teach patients the adaptive role of both positive and negative emotions, without the need to suppress or avoid them. Nevertheless, future studies based on mediational analyses will allow a better understanding of whether PA and NA would mediate the impact of treatment on therapy outcomes (ie, depression and anxiety). As predicted, both protocol modalities led to significantly greater reductions (relative to the WL) in depression and anxiety, as well as significant decreases in NA and increases in PA measures, suggesting that web-based transdiagnostic treatment was effective in treating EDs.

On the secondary outcomes (NEO FFI and EuroQoL-5D questionnaire, EQ-5D), the analysis also revealed a significant change from pre- to posttreatment. The effect sizes were medium for both variables in both intervention groups (except for Extraversion, which was smaller in the TIBP condition). In the WL group, small effect sizes were also found for Extraversion, showing that participants in this group experienced significantly lower levels of extraversion after the waiting period. Between-group comparisons revealed that participants who received the treatment scored better on the measures at posttreatment compared with the WL group. These findings contribute to the study of the *malleability* of neuroticism proposed by Barlow [86], who suggests that personality dimensions may be malleable over time. In this study, some strategies were proposed to modify PA (extraversion), and although significant effects were found between the two intervention groups and the WL group, further research is needed on this topic to explore the effect of this specific component on increasing well-being and PA. Along these lines, Barlow’s team conducted an RCT that evaluated changes in PA in cognitive-behavioral treatments for anxiety disorders, showing that PA is a malleable construct and can be influenced by CBT [87].

The second aim of this study was to assess the effects of the component designed to upregulate PA, with the hypothesis that the TIBP+PA condition would significantly outperform the TIBP condition on the PA measures. This study found no significant differences between the two treatment conditions on PA measures. This may be due to the fact that participants improved throughout the course of the treatment, decreasing negative symptoms and increasing their PA, even if they had not seen the last four modules with the specific component to upregulate PA. Therefore, future studies will be needed to reach a deeper understanding of the relationship between treatments that include PA components and changes in PA measures.

Another objective of the study was to study patients’ acceptance of the program developed to apply the treatment protocol over the internet with minimal support from clinicians. The overall expectations and satisfaction expressed by the participants in the two intervention groups were high, and both protocols were well accepted. However, the analysis revealed higher levels of expectations and satisfaction in the TIBP+PA condition. This may be due to the number of modules included in this condition (16) in comparison with the modules in the TIBP condition (12), although participants in the intervention groups did not know the content of those four additional modules. This result may be influenced by the role of expectations before and during a web-based psychological intervention, which is consistent with the literature showing that treatment expectations became more favorable over time [88]. Regarding the reliable change index, significant improvements were found in the two treatment conditions compared with the WL group. Overall, participants who received the transdiagnostic internet-based interventions showed higher recovery percentages and less deterioration compared with the WL group.

This study had a dropout rate of 38.9% (86/216). Some studies in the literature have indicated dropout rates of approximately 30% in computerized CBT programs [47]. However, this issue deserves special attention because it depends on how it is conceptualized. On the one hand, the definition of attrition differs and can be understood as premature termination [89], (non)persistence [90], (non)adherence, or the extent to which an individual is exposed to the content of an intervention [91]. In this study, only participants who completed all the treatment modules were considered completers, whereas the rest were treated as dropouts, although in other studies, only participants who completed a percentage of the modules were considered completers [92,93]. On the other hand, adherence to internet-based interventions is associated with the type of guidance, achieving 28% nonadherence when therapist-guided, and 38% when administrative support is given [94]. This study combined human and ICT support, reaching a 36.8% (53/144) dropout rate in the treatment conditions; therefore, these results are not far from those found in the existing literature on internet-based interventions. However, further research on attrition is needed to better design internet-based treatments and increase retention. The existing literature has indicated an important relationship between the support provided to the participants and adherence to the program. Indeed, this aspect has great relevance in the literature on internet-based interventions [95,96]. Therefore, if this type of intervention is carried out on a large scale in the community, as in this study, a fundamental factor to consider is the type and amount of support to provide, which would be consistent with the study of the balance between the benefits and resources involved in providing human support in internet-based interventions. Technologies play a central role in this aspect because part of the support can be provided automatically through technological devices. In an attempt to understand this issue, our research group conducted a qualitative study to determine why patients dropped out of the web-based transdiagnostic program for EDs (TIBP) [97], with the results emphasizing the lack of individualization of the treatment or the lack of support (ie, the lack of affective and personal contact with the therapist). In this regard, future studies should develop personalized treatments to address patients’ specific needs and increase adherence rates. There are some useful strategies for personalizing treatments, such as selecting certain treatment components to better fit the patient’s symptoms or lowering the number of sessions required to successfully treat an individual’s symptoms. One strategy implies personalizing the treatment to a specific presentation, that is, by selecting the treatment components that best fit the specific set of symptoms or *weaknesses* shown by each patient [10], thereby lowering the number of sessions required to successfully treat an individual’s symptoms. Another example is the study by Carl et al [98]. In this study, the authors presented a module for the regulation of PA that can be added to the treatment once they have completed the UP modules, thus personalizing the treatment for patients who show deficits in positive emotions at posttreatment.

This study has several strengths. First, it presents a novel focus in the field of transdiagnostic treatments. To the best of our knowledge, this is the first study of a transdiagnostic internet-based treatment for EDs with a specific component to upregulate PA. Overall, the findings indicate that EDs can be effectively treated with a transdiagnostic intervention via the internet, in addition to improving depression, anxiety, and quality of life measures. Regarding PA measures, promising effects were found, but more research is needed to study the role of positive emotions in the construction of psychological strengths [99,100] from a transdiagnostic perspective [42,101]. Moreover, this study included a large sample of people from a community sample, representing a heterogeneous population with EDs that does not receive primary or specialized care, with or without the presence of comorbidities. Thus, the transdiagnostic protocol represents a successful approach to the treatment of multiple disorders in a parsimonious manner [102]. Participants in the study seemed to be interested in the use of adaptive emotion regulation strategies, regardless of whether they were related to their own difficulty, which is the basis of transdiagnostic proposals. These interventions emphasize the essential processes underlying different disorders and the use of core *higher-order* strategies that eliminate the need for multiple diagnosis-specific manuals [103]. Moreover, the internet-based format of this transdiagnostic protocol facilitates the availability and administration of the program to provide support to anyone in need.

### Limitations

This study also has some limitations. The most important is the different number of modules in the two protocols (TIBP condition: 12 modules; TIBP+PA condition: 16 modules). The TIBP condition had more time between the last module and postassessment than the TIBP+PA condition. However, the TIBP+PA condition had more modules. Postassessment took place at the end of module 12 or 16. These aspects may have influenced the results. In an attempt to control this, equal time (ie, a maximum period of 18 weeks from randomization to posttreatment evaluation) was given to all participants to allow them to use the program as much as they desired throughout the whole process. However, future studies should show that the differential effect of the PA component is not simply because of the larger number of modules in the protocol. This leads to the importance of benchmarking based on previous transdiagnostic internet-based interventions with regard to effect sizes or the length of these interventions. This study coincides with previous similar transdiagnostic interventions that also obtained large effect sizes for depression (*g*=0.84) and medium effect sizes for anxiety (*g*=0.78), with a treatment length ranging from 6 to 10 sessions [18]. Furthermore, focusing on PA measures, it is important to mention that this study obtained larger effect sizes for PA, both for within- and between- comparisons (TIBP+PA, *d*=1.27; TIBP+PA vs WL, *d*=0.90) than other transdiagnostic interventions [13,14,50-53]. Another limitation of the study, shared with other transdiagnostic interventions, is that although they are called transdiagnostic treatments, they are based on discrete diagnostic categories (ie, DSM-IV). Future research should study the mechanistically transdiagnostic principles, that is, the underlying mechanisms that account for the occurrence of specific symptoms to include them in both assessments and transdiagnostic interventions. Some examples of transdiagnostic mechanisms that have been found to play a fundamental role in EDs are intolerance to uncertainty [104], rumination [105], perfectionism [106], or thought suppression [107]. Among these processes, neuroticism has been strongly associated with both anxiety and depressive disorders [86,108]. In addition, attrition rates were higher in both treatment conditions (TIBP: 26/71, 37%; TIBP+PA: 27/73, 37%) than in the WL condition (17/72, 24%). However, attrition rates of 30% to 35% are commonly observed in internet-based interventions [47]. Furthermore, although we consider that the sample of this study is quite representative, future research should focus on improving the mental health of less reachable groups such as older people, people with low income or lower educational level, and/or people residing outside urban areas (ie, rural populations). Moreover, it would also be important to collect data on certain demographic variables such as race, ethnicity, and sexual orientation in future trials. Transdiagnostic treatments that are socioculturally adapted for such groups could be a potential solution to effectively reach a larger number of people in need of psychological help.

Finally, follow-up data were not included in this study because we are still in the recruiting process, and we wanted to promptly provide the results because of the importance of the issue. The long-term effects of this intervention will be presented in further research.

### Conclusions

In summary, the results show the efficacy of a transdiagnostic internet-based psychological treatment protocol for individuals from a community sample with EDs. These findings show that this web-based transdiagnostic treatment improved the clinical situation of the participants, providing them with tools and strategies to face problems and difficulties more effectively. Future lines of research should carry out dismantling designs to determine the active components of the protocol, especially the contribution of the PA modules, and analyze the effectiveness of web-based treatment in other populations, such as primary care centers. Furthermore, the existing techniques and strategies to improve PA require further study to determine which ones are more effective and should be included as specific components to upregulate PA in current psychological interventions. This study includes some specific strategies to promote psychological strengths and enhance positive mood. However, it is important for future studies to explore more deeply the effect of these and other strategies on patients with EDs to directly build positive resources to counteract NA. Undoubtedly, future research will have to determine whether it is beneficial to include these components designed to enhance PA, which components are necessary for whom, and how they should be applied.

Furthermore, future research should focus on the possibility of developing treatment components aimed at altering, modifying, or varying vulnerability, a key aspect of transdiagnostic perspectives. This might be possible with strategies for modifying PA, but there are other fundamental factors that influence mental health and well-being. For example, a large body of literature has highlighted the importance of accurate perceptions of reality in psychological health, such as positive illusions or positive self-evaluations, perceptions of control or mastery, and unrealistic optimism [109,110]. In addition, there is evidence for the relationship between psychological flexibility and well-being, suggesting that being psychologically flexible has benefits for executive functioning, default mental states, and personality dimensions such as neuroticism [111]. Psychological flexibility has been considered a protective factor in improving physical health, mental health, and well-being [112]. Hence, there is growing interest in constructs such as pragmatic prospection, that is, thinking about a future with desired outcomes and avoiding undesired ones [113] or openness to the future characterized by PA toward the future [114]. This body of knowledge opens up the possibility of finding new strategies to improve the efficiency and effectiveness of future transdiagnostic treatment protocols for EDs as a way to more effectively address temperament vulnerabilities, that is, the core aspects of these disorders.

## Appendix

Multimedia Appendix 1 Description of the positive affect modules.

Multimedia Appendix 2 CONSORT-eHEALTH checklist (V 1.6.1).

## Abbreviations

AG: agoraphobia
BA: behavioral activation
BAI: Beck Anxiety Inventory
BDI-II: Beck Depression Inventory, Second Edition
BI: behavioral inhibition
CBT: cognitive behavioral therapy
CONSORT: Consolidated Standards of Reporting Trials
DD: dysthymic disorder
DSM-IV: Diagnostic and Statistical Manual of Mental Disorders, Fourth Edition
E: extraversion
EBT: evidence-based psychological treatment
ED: emotional disorder
GAD: generalized anxiety disorder
ICT: information and communication technology
ITT: intention-to-treat
MDD: major depressive disorder
MNAR: missing not at random
N: neuroticism
NA: negative affect or negative affectivity
NEO FFI: NEO Five Factor Inventory
PA: positive affect
PANAS: Positive and Negative Affect Schedule
PD: panic disorder
RCI: reliable change index
RCT: randomized controlled trial
SAD: social anxiety disorder
TIBP: transdiagnostic internet-based protocol
UP: Unified Protocol
WL: waiting list
