# Supplementary material for: Negative and Positive Affect Regulation in a Transdiagnostic Internet-Based Protocol for Emotional Disorders: Randomized Controlled Trial
Source: J Med Internet Res. 2021 Feb 1;23(2):e21335. doi: 10.2196/21335 (PMC7884218; doi:10.2196/21335)
Supplement: Multimedia Appendix 1 [file jmir_v23i2e21335_app1.doc]

Appendix 1. Description of the positive affect modules

| Modules | Objectives | Strategies | Empirical source |
| --- | --- | --- | --- |
| M.12 Learning to move on | - To teach the relationship between inactivity and emotional distress and how being active can improve emotional well-being  - To teach the importance of short- and long-term significant life goals | To complete a diary of daily activities. monitoring both the level of satisfaction with the activities the patient is involved in during the day and to what extent they are linked to his or her personal goals and values. | Behavioral activation (Lejuez et al., 2001 [1]) |
| M.13 Learning to enjoy | To teach about the role of positive emotions in life and how to generate and maintain them | - The importance of smiling (Duchenne smile)  - The importance of “savoring”  - The importance of the “small things” | - Fredrickson’s Broaden-and-Build Theory (Fredrickson, 2001 [2])  - The Duchenne smile (Ekman et al., 1990 [3]; Soussignan, 2002 [4]; Miles and Johnston, 2007 [5])  - Savoring (Bryant and Veroff, 2007 [6]) |
| M.14 Learning to live | - To teach the concept of psychological strengths and how can we achieve psychological well-being  - To teach that mistakes are opportunities to learn about the strengths we wish to improve  - Identification of personal values and life goals; Identification of meaningful activities linked to personal values | - To promote own psychological strengths  - The concept and dimensions of well-being  - Select and record activities linked to values and significant areas and goals in life | - Psychological strengths (Peterson and Seligman, 2004 [7])  - Dimensions of well-being (Ryff, 1995, 2014 [8-9]) |
| M.15 Living and learning | - To promote emotions linked to well-being such as gratitude and positive emotional functioning, curiosity and life satisfaction  - To Identify and maintain wellbeing episodes | - Strategies to develop our strengths  - Exercises with gratitude, curiosity and hope | - Gratitude (Seligman et al., 2005 [10])  - Hope (Sheldon and Lyubomirsky, 2006 [11])  - Well-Being Therapy (Fava, 1999 [12]) |

References

1. Lejuez C, Hopko D, Hopko S. A Brief Behavioral Activation Treatment for Depression. *Behav Modif*. 2001;25(2):255-286. doi:10.1177/0145445501252005

2. Fredrickson B. The role of positive emotions in positive psychology: The broaden-and-build theory of positive emotions. *American Psychologist*. 2001;56(3):218-226. doi:10.1037/0003-066x.56.3.218

3. Ekman P, Davidson RJ, Friesen WV. The Duchenne smile: emotional expression and brain physiology: II. *Journal of personality and social psychology*. 1990; 58(2): 342.

4. Soussignan R. Duchenne smile, emotional experience, and autonomic reactivity: a test of the facial feedback hypothesis. *Emotion*. 2002;2(1): 52.

5. Miles L, Johnston L. Detecting happiness: Perceiver sensitivity to enjoyment and non-enjoyment smiles. *Journal of Nonverbal Behavior*. 2007;31(4): 259-275.

6. Bryant FB, Veroff J. *Savoring: A new model of positive experience*. Mahwah, NJ: Erlbaum; 2007.

7. Peterson C, Seligman EP. *Character strengths and virtues: A handbook and classification*. Oxford, UK: Oxford University Press; 2004.

8. Ryff CD. The structure of psychological well-being revisited. *Journal of Personality and Social Psychology*. 1995;69: 719-727.

9. Ryff CD. Psychological well-being revisited: Advances in the science and practice of eudaimonia. *Psychotherapy and Psychosomatics*. 2014;83:10-28.

10. Seligman ME, Steen TA, Park N, Peterson C. Positive psychology progress: Empirical validation of interventions. *American Psychologist*. 2005:60:410-421.

11. Sheldon KM, Lyubomirsky S. How to increase and sustain positive emotion: The effects of expressing gratitude and visualizing best possible selves. *The Journal of Positive Psychology*. 2006;1:73-82.

12. Fava G. Well-Being Therapy: Conceptual and Technical Issues. *Psychother Psychosom*. 1999;68(4):171-179. doi:10.1159/000012329
